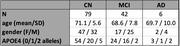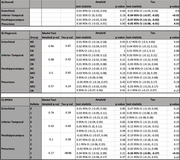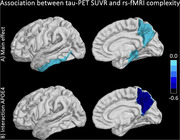# Effect of diagnostic and genetic status on the relationship between rs‐fMRI complexity and tau & amyloid PET in Alzheimer’s disease

**DOI:** 10.1002/alz.087143

**Published:** 2025-01-09

**Authors:** Kay Jann, Steven Cen, Mariella I Santos, Dilmini Wijesinghe, Ru Zhang, John M Ringman, Danny JJ Wang

**Affiliations:** ^1^ Mark and Mary Stevens Neuroimaging and Informatics Institute, University of Southern California, Los Angeles, CA USA; ^2^ Keck School of Medicine, University of Southern California, Los Angeles, CA USA; ^3^ Memory and Aging Center, University of Southern California, Los Angeles, CA USA; ^4^ Alzheimer’s Disease Research Center, Keck School of Medicine, University of Southern California, Los Angeles, CA USA

## Abstract

**Background:**

Non‐linear statistical analyses on resting‐state fMRI (rs‐fMRI) using complexity measures have demonstrated progressive decline in complexity from cognitively normal subjects (CN) to patients with Mild Cognitive Impairment (MCI) and patients with Alzheimer’s disease (AD). While complexity has been shown to be negatively associated with tau‐PET, the association with amyloid or effects of genetic characteristics (APOE4) remains unknown.

**Method:**

From the Alzheimer’s Disease Neuroimaging Initiative (ADNI3) we identified participants with tabulated SUVR values for amyloid and tau as well as one resting state fMRI scan for the same visit. The rs‐fMRI complexity was calculated as Multiscale Sample Entropy (MSE) (r=0.5, m=2, scale=6). SUVR values from precuneus, parahippocampal, inferior temporal and entorhinal regions were used in a multivariate generalized linear model to investigate the adjusted independent effects to corresponding rs‐fMRI complexity. Whether such effects were modified by either diagnosis (CN, MCI, AD) or APOE4 status (# alleles) were tested using the interaction terms in the multivariate model. Age and gender were controlled for all models.

**Result:**

The final cohort consisted of 127 subjects (Table 1). We observed statistically significant negative associations between complexity and tau in parahippocampus, inferior temporal gyrus and precuneus (Table 2A). Diagnostic status does not modify these associations, however, APOE4 status showed a statistically significant interaction effect for tau and complexity in precuneus (Table 2C). For amyloid no associations nor interaction effects were found (Table 2B).

**Conclusion:**

Our study confirmed previously reported statistically significant inverse relationship between rs‐fMRI complexity and tau‐PET, which is indicative of disfunction of neuronal signaling in the presence of tauopathy. While diagnostic classification showed no effects, the APOE4 genetic risk had a strong modifying effect leading to a stronger negative relationship between tau‐PET and fMRI‐complexity. The null‐finding for amyloid was expected, since, while the presence of amyloid increases risk for cognitive decline, it is the occurrence of tau that leads to cognitive decline and neurodegeneration. Overall, genetic risk potentially increases the prevalence of amyloid in this cohort and consequentially leads to a cumulative and more pronounced decrease in rs‐fMRI complexity in the presence of tau.